# Accidental Subcutaneous Remifentanil Infusion as a Cause of Delayed Awakening after Craniotomy

**DOI:** 10.1155/2011/919067

**Published:** 2011-11-03

**Authors:** Alexander Wolfson, Cephas Swamidoss

**Affiliations:** ^1^Department of Anesthesiology, University Medical Center at Princeton, 253 Witherspoon Street, Princeton, NJ 08540, USA; ^2^Department of Anesthesiology, Hospital for Special Surgery, 535 East 70th Street, New York, NY 10021, USA

## Abstract

We report a case of accidental subcutaneous infusion of remifentanil as a cause of delayed awakening after a craniotomy.

## 1. Case Report

A 60-year-old male (5′5′′, 73.7 kg) presented for resection of a left acoustic neuroma via a left retromastoid suboccipital craniotomy. His past medical history was significant only for hypertension, which was well controlled with beta-blockade. His physical examination and laboratory workup did not reveal any abnormalities other than decreased hearing and the presence of an acoustic neuroma on imaging studies. He arrived in the operating room with a 20-gauge intravenous catheter in the left hand at heplock. A second 18-gauge peripheral intravenous catheter (IV) was placed in the patient's right hand. After standard monitoring was applied according to recommendations of the American Society of Anesthesiologists, Midazolam 2 milligrams (mg) was administered. General anesthesia was induced with Thiopental 375 mg, Fentanyl 150 micrograms (mcg), Lidocaine 60 mg, and *cis*-atracurium 10 mg. All intravenous medications for induction were administered via the 18 g intravenous catheter. Anesthesia was maintained with 0.5 MAC Desflurane in Oxygen/Nitrous Oxide 1/1.5 liters per minutes and Remifentanil (“Ultiva,” Abbott Laboratories, North Chicago, IL 60064, USA) at 0.15 mcg∗kg^−1^∗min^−1^ administered via the 20 g IV catheter. It is important to note that throughout the entire procedure the only medication administered through the 20 g IV catheter was the Remifentanil infusion. A neurology team was present to monitor brainstem auditory evoked potentials (BAERs) during resection of the neuroma. Vancomycin 1 gm IV, Mannitol 70 gm IV, Dexamethasone 10 mg IV, and Furosemide 10 mg IV were administered via the 18 g IV over recommended time periods. Approximately 45 minutes after surgical incision, the patient's anesthetic requirements began to rise such that within 2 hours of incision, he required 1 MAC of Desflurane in addition to Nitrous Oxide and Remifentanil in order to maintain homeostasis. At approximately 2.5 hours of operating time, the patient became increasingly tachycardic and was treated with 10 mg of Metoprolol over the next 10 minutes. Train of four (TOF) was continuously monitored throughout the case, and the trend of four (TOF) ratio was noted to be 4/4 at 60 minutes after the initial, and only, dose of *cis*-atracurium. The patient was allowed to resume spontaneous ventilation 3 hours after induction at which time the tidal volume was 300–400 milliliter (mL), with a respiratory rate of 18-19 breaths per minute. Morphine, 3 mg, IV was administered via the 18 g catheter, while the Remifentanil infusion remained at 0.15 mcg∗kg^−1^∗min^−1^. Remifentanil was discontinued 12 minutes after administration of Morphine, as the patient was breathing at a rate of 14–16 breaths per minute and achieving tidal volumes of 400–500 mL. The total surgical time was a little over 3 hours. Surgery was completed 10 minutes after discontinuation of Nitrous Oxide, 15 minutes after Desflurane, 20 minutes after Remifentanil, and 32 minutes after administration of Morphine. When the surgical drapes were taken down, the 20 g IV was noted to be infiltrated, and the patient's hand had become swollen. At this point, it became apparent that the Remifentanil had been infusing subcutaneously for several hours. The presence of adequate circulatory flow to the left arm was confirmed by palpation and Doppler. The patient maintained adequate ventilation and hemodynamic stability but did not exhibit any signs of wakefulness. He did not exhibit any response to painful stimuli. At this time, 0.04 mg of Naloxone was administered intravenously, and the patient began to follow verbal commands. After meeting extubation criteria, 45 minutes after the end of surgery, the trachea was extubated. Extubation criteria used included hemodynamic stability, eye opening, ability to follow commands, strong grip, more than 5-second headlift, swallowing, and purposeful action. The patient was transported to the recovery room with monitors and supplemental nasal cannula oxygen. After his arrival in the recovery room, he required a second dose of Naloxone, as his level of consciousness began to decrease. After this time, he did not exhibit any further signs of sedation. Of note, he did not complain of pain at the surgical site for several hours after being received in the recovery room. The total intraoperative fluids were 1000 mL NS, 150 mL estimated blood loss (EBL), and 2100 mL urine output (UOP). Normothermia to 37 degrees Celsius was maintained throughout the case with the use of warming blankets and fluid warmers. The subcutaneous infiltration in the hand resulted in mild blistering but was treated with warm compresses and resolved over the next 3 days without sequelae. The patient was discharged home and had uneventful postoperative visits with the surgical team.

## 2. Discussion

Delayed awakening is a source of concern in the postoperative neurosurgical setting. This case provides an example of a neurosurgical patient with delayed emergence after a relatively short general anesthetic. The differential for this phenomenon is broad, but for the purposes of this discussion, it includes iatrogenic neurosurgical intracranial pathology, metabolic derangements, and pharmacologic causes including residual benzodiazepine, muscle relaxant, inhalational agent, and opioid effects [1, 2]. 

Although difficult to assess without imaging studies or other cortical function tests done immediately after resection, the possibility of intracranial pathology remains high on the differential. In this particular case, the operative time was short, and the surgery was uneventful. In addition, there was minimal blood loss, and brainstem auditory evoked potentials were appropriately monitored by neurologists throughout the neuroma resection as it surrounded the left eighth cranial nerve [3–5]. Desflurane, a well-known inhibitor of BAERs, was kept under one MAC to allow for intraoperative monitoring and was only increased upon completion of the actual nerve resection [6]. Follow-up imaging studies did not reveal any evidence of thrombotic, hemorrhagic, or other intracranial pathology. In addition, the use of beta blockade has been shown to blunt postoperative hyperemia and its deleterious effect following intracranial surgery [7]. Once fully awake the patient did not exhibit any focal neurological findings.

Metabolic derangements can also play a role in delayed awakening. However, the patient did not have a history of an endocrine disorder such as diabetes mellitus or hypothyroidism, nor did he have renal or liver disease. Despite the use of Mannitol and Furosemide, no postoperative electrolyte abnormalities were noted in the recovery room. 

Pharmacologic causes of delayed awakening from general anesthesia are numerous. In this case, all anesthetic agents used for induction and maintenance were chosen for their rapid metabolism and short half-life. A relatively healthy 60-year-old male who received Midazolam, Thiopental, Desflurane, Nitrous Oxide, and Remifentanil during his operation should not have delayed awakening. At the conclusion of surgery, Desflurane and Nitrous Oxide levels were undetectable by end-tidal spectrometry. A 2 mg dose of Midazolam is also unlikely to explain this patient's perioperative course. Morphine administered toward the end of the procedure was titrated to the patient's respiratory parameters, and it would seem implausible that a 3 mg dose could cause such an extensive delay. Residual muscle relaxation is also an unlikely culprit in this case as the patient was able to maintain adequate spontaneous ventilation prior to the end of surgery and throughout his immediate postoperative course.

In light of the improbability of other causes for delayed awakening, we suggest that it was the accidental subcutaneous Remifentanil depot that served as the cause of delayed emergence in this case. Remifentanil was chosen as the analgesic component of our anesthetic because its use has been shown to decrease MAC requirements of inhalational agents while allowing for rapid recovery and cardiovascular and neurological stability [8–12]. 

Remifentanil is a congener of the fentanyl family of opioids that was approved for use as a supplement to general anesthesia by the US Food and Drug Administration in 1996 [13]. It has since become widely used in pediatric, ophthalmic, head and neck, and neuroanesthesia [14].

Remifentanil, the hydrochloride salt of 3-[4-methoxy-carbonyl-4-[(1-oxopropyl)phenylamino]-1-piperidine] propanoic acid methyl ester, is commercially available in a powder form and must be reconstituted into a solution for administration [15]. Its metabolism is rapid and clinical use depends on infusion-based dosing. Any interruption of the infusion such as an accidental disconnection, pump malfunction, empty drug reservoir, or as in this case infiltration of the IV, leads to a rapid decrease in the anesthetic and analgesic effects requiring further escalation in other anesthetic agents [16].

The pharmacokinetics of remifentanil have been described in numerous studies and have been shown to be more closely related to lean body mass than to total body weight [17]. Several studies have investigated the pharmacodynamics of Remifentanil and found it to be 20–40 times more potent than Alfentanil and nearly equipotent with Fentanyl [18, 19]. Remifentanil is rapidly metabolized by nonspecific tissue esterases in blood and other tissues including the brain, liver, placenta, and GI tract [20–22]. However, we found no study investigating the existence of enzymatic degradation of Remifentanil in subcutaneous tissues. Also important to note is that Remifentanil is not a substrate for pseudocholinesterase and, therefore, is not influenced by pseudocholinesterase deficiency [23]. Furthermore, renal and hepatic function has been shown not to influence duration of its clinical action [24, 25]. However, hypothermia has been shown to decrease enzymatic function of plasma esterases by about 20% potentially leading to accumulation of Remifentanil [26]. 

In the case presented, there were no surgical complications, blood loss and fluid administration were not excessive, hepatic and renal functions were not compromised, hypo- and hypertensions were carefully avoided, and normothermia was maintained throughout. The unanticipated need for increasing intraoperative anesthetics was met with increasing doses of Desflurane, Metoprolol, and Morphine. The subcutaneous Remifentanil, serving as a depot, caused delayed awakening and arguably provided postoperative analgesia. This patient appropriately regained consciousness following administration of opioid antagonists and did not require any further pain relief in the immediate postoperative period.

Subcutaneous administration of anesthetic agents has been the subject of multiple investigations [27, 28]. However, we were only able to find one other mention of subcutaneous Remifentanil administration in the literature [29]. Curiously enough, there are several studies looking at the behavior of subcutaneously administered Morphine and its metabolites [27, 28, 30]. One of the more interesting findings of these studies is that the metabolism and clinical actions of subcutaneously administered opioids are extremely unpredictable and are associated with a tremendous amount of interuser variability [27, 30]. Subcutaneously administered insulin has been shown to act as a slow and steady infusion with speed of absorption affected by subcutaneous blood flow and location of the depot on the patient [31]. Unfortunately, in reviewing the published kinetics models of Remifentanil, we were unable to find any references to its behavior when administered subcutaneously [14, 16–21, 32–34]. This raises the possibility that a drug such as Remifentanil, with an extremely favorable metabolic profile when administered intravenously, does not behave in the same way when administered subcutaneously and may have prolonged opioid effects. Therefore, caution should be exercised when Remifentanil is administered as an infusion during operations where the IV site is not clearly visible. 

We have described a case of an infiltrated IV leading to a subcutaneous depot of Remifentanil and prolonging emergence after a relatively quick and otherwise uneventful neurosurgical procedure. Unfortunately, we did not have the capability to immediately obtain quantitative Remifentanil or its by-product levels in our laboratory. We also did not yet have the opportunity to investigate the existence of subcutaneous enzymatic degradation of Remifentanil and its potential clinical uses as a subcutaneous depot similar to that of other narcotics.
